# CD70: An emerging target for integrated cancer diagnosis and therapy

**DOI:** 10.1002/ctm2.70400

**Published:** 2025-07-09

**Authors:** Jiatao Hu, Jinxin Li, Bo Yang, Siyi Wang, Yi Bao, Yiren Yang, Kun Qiao, Fei Guo, Xinxin Gan, Linhui Wang

**Affiliations:** ^1^ Department of Urology, Changhai Hospital Naval Medical University Shanghai China; ^2^ Department of Urology, Eastern Hepatobiliary Surgery Hospital Naval Medical University Shanghai China; ^3^ School of Materials Science and Engineering, University of Shanghai for Science and Technology Shanghai China

**Keywords:** CD70, immuno‐PET/CT, CAR‐T, combination therapy, targeted therapy

## Abstract

CD70, which is a ligand belonging to tumour necrosis factors, exhibits aberrant overexpression among multiple hematologic and solid malignancies and has drawn extensive research enthusiasm as a target to treat and diagnose cancers. CD70‐targeted immuno‐PET/CT is an emerging approach for cancer diagnosis, with the potential to improve tumour detection, assist disease staging, and monitor therapeutic response. CD70‐targeted therapeutic approaches, involving antibody–drug conjugates, CAR‐T, CAR‐NK, and monoclonal antibodies, have shown encouraging activity in preclinical models and preliminary signals of efficacy in early clinical studies. In addition to these monotherapies, increasing efforts have focused on rational combination strategies that aim to enhance antitumor efficacy, reverse immune suppression, and overcome resistance. This review comprehensively summarizes the diagnostic and therapeutic advances in CD70‐targeted strategies, discusses ongoing barriers, and outlines future directions to advance CD70‐targeted imaging and therapeutic strategies through mechanistic research and clinical translation.

**Key points:**

- CD70 is aberrantly overexpressed in diverse malignancies with limited normal tissue expression.
- CD70‐targeted immuno‐PET/CT improves tumour detection, staging, and treatment monitoring.
- Multiple CD70‐targeted therapies, including CAR‐T and CAR‐NK, are under active investigation.
- Rational combination strategies are emerging to enhance antitumor efficacy.

## Graphical abstract

CD70 is aberrantly overexpressed in diverse tumours with limited normal tissue expression, making it a compelling diagnostic and therapeutic target. Immuno‐PET/CT facilitates tumour detection, staging, and treatment monitoring. Multiple CD70‐targeted therapies are under early evaluation. Rational combination strategies are emerging to enhance antitumor efficacy.

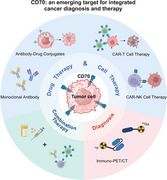

## 1 INTRODUCTION

The rising incidence and mortality of cancer have posed persistent challenges to public health worldwide.^1^ Although surgery, chemotherapy, and radiotherapy remain foundational treatment modalities, their efficacy in advanced malignancies is often compromised by patient intolerance and tumor resistance.^2^, ^3^ Furthermore, the detection and staging diagnosis of tumours, which are essential for precision therapy, still face several limitations. Traditional imaging methods, including MRI, ^18^F‐FDG PET/CT, and CT, may show limited diagnostic and prognostic utility in certain tumours including renal cell carcinoma (RCC).^4^, ^5^, ^6^ Some lesions show inadequate ^18^F‐FDG uptake, and distinguishing inflammation from tumour may be challenging, resulting in missed or incorrect diagnoses.^7^, ^8^ These limitations underscore the necessity of identifying novel molecular targets and designing strategies that can simultaneously improve diagnostic accuracy and therapeutic efficacy.

Recent advances in immunotherapeutic and molecularly targeted approaches have expanded therapeutic options for cancer sufferers experiencing drug resistance or disease recurrence.^9^ For instance, the therapeutic landscape of oncology has been reshaped by clinical advances in chimeric antigen receptor T cell (CAR‐T) and immune checkpoint inhibitors (ICIs) immunotherapies.^10^, ^11^ However, the applicability of targeted and immune therapies still faces numerous challenges, as certain patients exhibit limited responsiveness or eventually acquire therapeutic resistance, often alongside pronounced immune‐related adverse effects.^12^, ^13^ Accordingly, selecting suitable molecular targets is pivotal to balancing therapeutic effectiveness with patient safety.^14^ Meanwhile, the integration of precise tumour diagnosis and immunotherapy is still in the early stages of exploration.^15^ One major challenge lies in utilizing molecular imaging to guide patient stratification for targeted immunotherapy and to enable real‐time evaluation of treatment responses.^16^

CD70 (CD27L) is a ligand for CD27, which belongs to tumour necrosis factor. Under normal circumstances, CD70 exhibits confined and transient expression in B, T, and dendritic cells upon their activation.^17^, ^18^, ^19^ Interacting with CD27, CD70 exerts key regulatory effects on the fate and functionality of T cells, promoting their proliferation, survival, and the development of effector functions, while also supporting memory T cell long‐term maintenance and differentiation.^20^ In contrast, under pathological conditions, CD70 is abnormally overexpressed in various hematologic and solid tumours.^21^, ^22^, ^23^, ^24^ Notably, recent pan‐cancer analyses leveraging integrated datasets from the databases of Human Protein Atlas (HPA), Genotype‐Tissue Expression Project (GTEx), Tumour Immune Single Cell Hub (TISCH), and Cancer Genome Atlas (TCGA) have systematically characterized CD70 expression across diverse malignancies.^25^, ^26^ These studies collectively assessed CD70 at the transcriptomic, proteomic, and single‐cell levels, demonstrating its aberrant upregulation in tumours involving RCC, mesothelioma, and diffuse large B‐cell lymphoma (DLBCL), while showing minimal or no expression among the majority of noncancerous tissues.^25^, ^26^ This heterogeneity suggests that distinct regulatory mechanisms may govern its aberrant expression across cancers. For instance, hypoxia‐inducible factors (HIFs) are stabilized because of impaired von Hippel–Lindau (VHL) activity to activate CD70 transcription within clear cell RCC (ccRCC) cells.^27^, ^28^ In nasopharyngeal carcinoma (NPC), the transcription of CD70 was found to be activated by NFKB2 upon infection with Epstein–Barr virus.^29^ Furthermore, during epithelial‐to‐mesenchymal transition (EMT) of non–small cell lung cancer (NSCLC) cells, particularly within drug‐tolerant persister ones associated with EGFR‐TKI resistance, CD70 is upregulated. This regulation is largely driven by the transcription factor ZEB1, and further enhanced by TGF‐β signalling and CD70 promoter hypomethylation.^30^, ^31^ CD70 overexpression within malignant cells is implicated in aggressive biological behaviours, including increased proliferation, invasiveness, stemness, EMT, therapeutic resistance, and other malignant features.^31^, ^32^, ^33^, ^34^ By interacting with its receptor presented by T cells, CD70 expressed on malignant cells could deplete T cells, trigger apoptotic effector T cell death, and facilitate regulatory T cell (Treg) accumulation, thereby creating an immunosuppressive microenvironment that enables tumours to evade immune surveillance.^20^, ^35^, ^36^

The remarkable differences in CD70 abundance between noncancerous and cancerous tissues render the molecule an evident utilization potential in diagnosing and treating various malignancies. Significant progress has been made in developing therapeutic strategies targeting CD70, including antibody–drug conjugates (ADCs), monoclonal antibodies, and engineered immunotherapeutic cells, including CAR‐NK and CAR‐T.^37^, ^38^, ^39^ Moreover, its application as a molecular imaging biomarker has also been extended to PET/CT, further expanding its clinical value in tumour diagnosis.^40^

**FIGURE 1:**
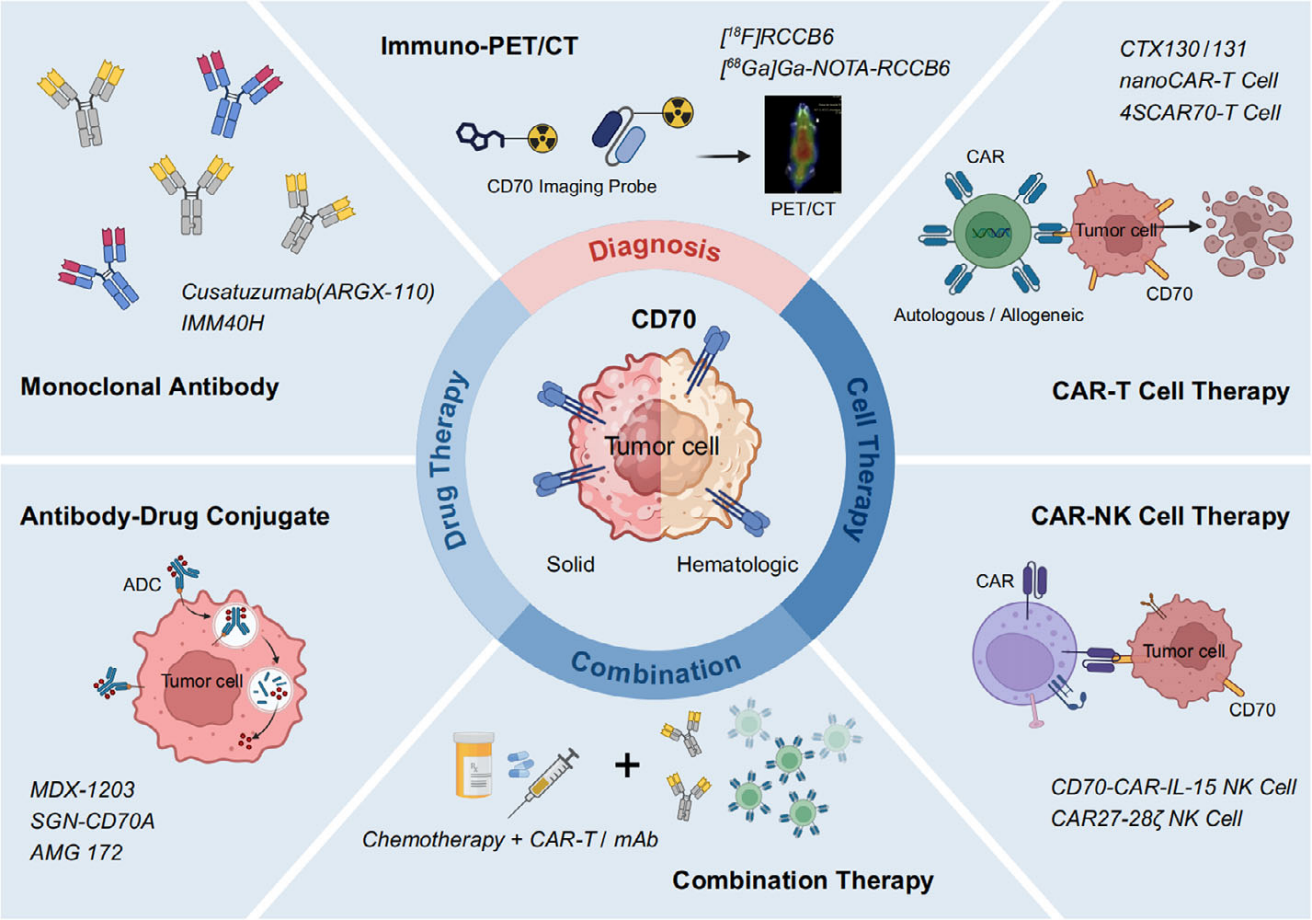
Overview of CD70‐targeted strategies in cancer diagnosis and therapy. CD70‐targeted immuno‐PET/CT has shown unique application potential in cancer diagnosis, highlighting its value as an emerging diagnostic tool. Additionally, various strategies targeting CD70 in solid and hematologic cancers are illustrated, including monoclonal antibodies (mAb), antibody‐drug conjugates, CAR‐T and CAR‐NK cell therapy. Combination therapy is receiving increasing attention for its potential to enhance therapeutic efficacy.

Herein, we comprehensively summarize recent progress in CD70‐targeted strategies across both solid and hematologic malignancies (Figure 1). By integrating developments in diagnostic imaging, treatment design, and combination strategies, it aims to offer perspectives to inform the rational design and clinical translation of CD70‐based diagnostic and therapeutic modalities.

## 2 THE ROLE OF CD70 IN CANCER DIAGNOSIS

Immuno‐PET/CT is a molecular imaging technique that utilizes radiolabeled molecular probes to target tumour‐associated antigens, demonstrating significant advantages in the precise diagnosis of tumours and postoperative monitoring.^41^ Recent developments in radiotracers targeting tumour stromal and surface markers, such as fibroblast activation protein (FAP) and Nectin‐4, have further expanded the application of immuno‐PET/CT beyond traditional markers, demonstrating excellent tumour visualization and potential utility for patient stratification.^42^, ^43^, ^44^, ^45^ The molecular probes used in existing CD70‐related research are mainly based on single‐domain antibodies (sdAb, i.e., nanobodies), combined with the NOTA chelator and labelled with the radionuclides ^18^F or ^68^Ga^40^, ^46^, ^47^, ^48^, ^49^ (Table 1). These probes exhibit high targeting specificity, stability, low immunogenicity, rapid imaging, and excellent safety.

**TABLE 1:** CD70‐targeted immuno‐PET/CT in preclinical and clinical studies.

Name	Cancer	Research type	Clinical trial identifier	Phase	PMID
[^18^F]RCCB6	Renal cancer, lymphoma	Clinical Trial	NCT06680089 NCT06148220	IIT	38480552 39043547 39243726
[^68^Ga]Ga‐NOTA‐RCCB6					39510586
[^68^Ga]Ga‐NOTA‐anti‐CD70 VHH	Renal cancer, etc.	Preclinical study	Not applicable	–	37093350

Abbreviations: CD, cluster of differentiation; IIT, investigator‐initiated trial.; NCT, National Clinical Trial; PET/CT, positron emission tomography/computed tomography.

**FIGURE 2:**
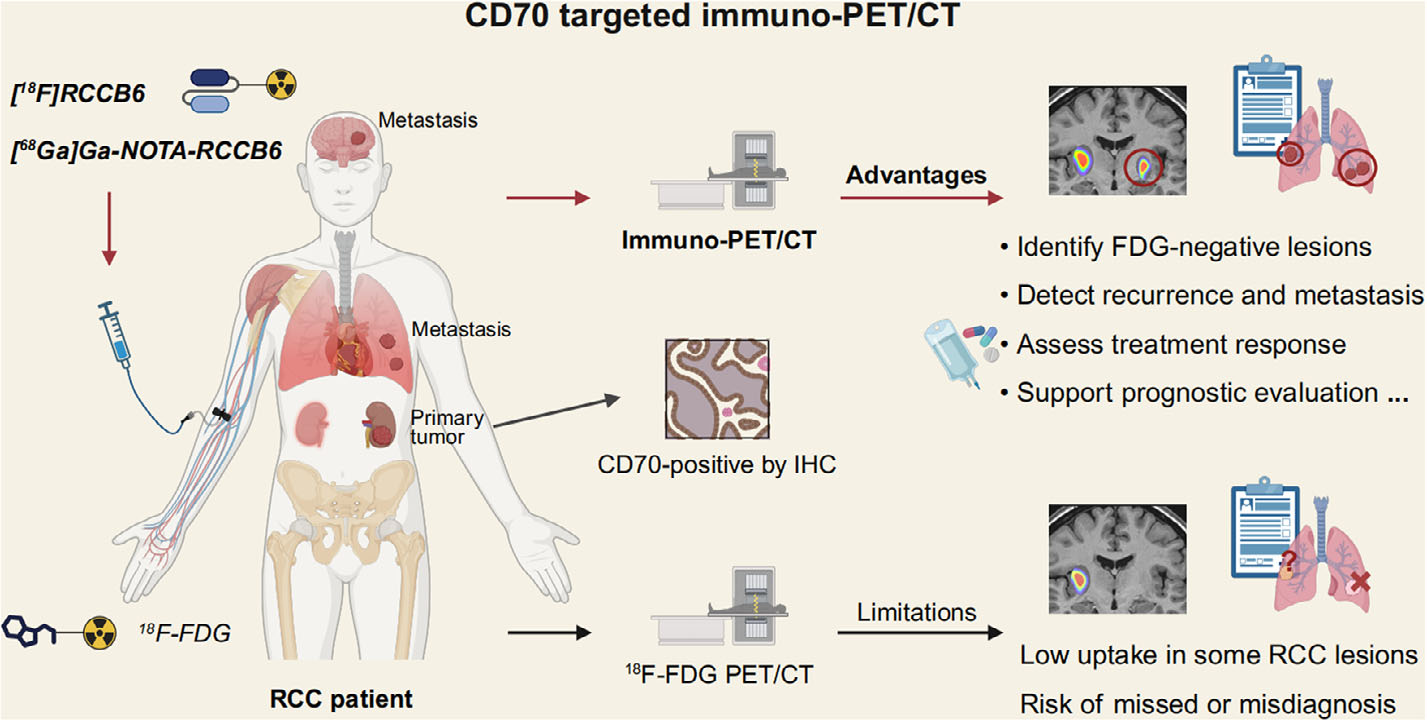
CD70‐targeted immuno‐PET/CT in RCC diagnosis. CD70‐targeted tracers ([^18^F]RCCB6 and [^68^Ga]Ga‐NOTA‐RCCB6) enable the detection of CD70‐positive primary and metastatic lesion in renal cell carcinoma (RCC), as confirmed by immunohistochemistry (IHC). Compared with CD70 immuno‐PET/CT, conventional ^18^F‐FDG PET/CT shows limited diagnostic efficacy due to low tracer uptake in some RCC lesions and poor specificity, which may lead to missed or incorrect diagnoses. CD70‐targeted immuno‐PET/CT also shows potential in treatment monitoring and disease progression assessment.

Given the frequent overexpression of CD70 in RCC, particularly in ccRCC, CD70‐targeted immuno‐PET/CT may help overcome the limitations of conventional ^18^F‐FDG PET/CT in RCC by addressing issues such as low tracer uptake in certain lesions and the challenge of distinguishing inflammation as well as benign and malignant tumours^6^, ^7^, ^8^ (Figure 2). A clinical study conducted by Wu and Zhou et al. (NCT06148220) evaluated two CD70‐targeted immuno‐PET/CT probes, [^18^F]RCCB6 and [^68^Ga]Ga‐NOTA‐RCCB6.^40^, ^47^, ^48^, ^49^ Notably, [^18^F]RCCB6 successfully detected a primary ccRCC lesion that was negative on ^18^F‐FDG PET/CT, with delayed imaging showing clear tracer accumulation and postoperative pathology confirming strong CD70 expression^47^. Beyond primary lesions, this study also reported the detection of multiple metastatic RCC lesions—including intracranial metastases—that were missed by conventional imaging, highlighting its potential in both primary and metastatic lesions detection^40^, ^48^, ^49^ (Figure 2). However, in one case of papillary RCC with lung and retroperitoneal metastases, [^68^Ga]Ga‐NOTA‐RCCB6 showed no uptake in metastatic lesions, while ^18^F‐FDG PET/CT yielded positive signals, suggesting that current CD70‐targeted tracers may have limited sensitivity in tumours with low CD70 expression.^49^ Furthermore, CD70‐targeted immuno‐PET/CT is also being explored for treatment response monitoring and long‐term follow‐up.^49^ Nevertheless, several limitations should be considered. CD70 expression in activated immune cells, nonneoplastic tissues, or certain non‐RCC tumours may contribute to false‐positive signals, especially under inflammatory or immune‐reactive conditions. Moreover, the currently available tracers exhibit high physiological renal uptake due to suboptimal pharmacokinetics, which may obscure the detection of primary RCC.^40^, ^47^

Future advancements should focus on optimizing the pharmacokinetics of CD70‐targeted tracers and developing alternative molecular tracers, including nucleic acid aptamers, peptides, and small‐molecule probes, to further improve targeting specificity and minimize off‐target effects.^50^, ^51^, ^52^ Although early clinical studies have shown promising specificity, comparative data with other molecular imaging targets, such as CAIX or PSMA, remain limited, warranting trials with larger sample sizes for validating CD70‐targeted immuno‐PET/CT's diagnostic utility across RCC subtypes and other malignancies characterized by CD70 overexpression.

## 3 CANCER THERAPEUTIC STRATEGIES TARGETING CD70

### 3.1 Monoclonal antibodies

Monoclonal antibodies, as one of the most basic and established targeted therapies, offer numerous advantages such as high purity, strong specificity, and good reproducibility, and are widely applied in fields such as oncology, haematological disorders, immunology, and infectious diseases.^53^, ^54^ They also form the foundation for many novel therapies, such as ADCs and CAR‐T.

Cusatuzumab (ARGX‐110) is the first CD70‐targeted monoclonal antibody developed by Argenx that is extensively studied and applied. It is a defucosylated IgG1 monoclonal antibody that restores systemic immunosurveillance for tumours through inactivating CD70.^55^ In addition, cusatuzumab directly kills CD70‐expressing tumour cells through enhanced antibody‐dependent cell‐mediated phagocytosis (ADCP), complement‐dependent cytotoxicity (CDC), and antibody‐dependent cell‐mediated cytotoxicity (ADCC).^55^ A phase I trial adopting a dose escalation scheme investigated cusatuzumab among 26 individuals suffering from late‐stage solid or hematologic malignancies, focusing on safety and early efficacy. All the participants displayed satisfactory tolerance to the drug, revealing neither dose‐limiting toxicities nor maximally tolerated dose. Stable disease (SD) was observed in 53.8% of participants, suggesting acceptable tolerability and initial antitumour potential.^56^ A phase I/II investigation (NCT01813539) recruited individuals suffering from refractory or recurrent cutaneous T‐cell lymphoma (CTCL). Twenty‐seven CD70‐positive patients were enrolled, comprising 9, 14, and 4 participants, respectively suffering from Sézary syndrome (SS), mycosis fungoides (MF), and additional subtypes of the disease.^57^ Patients received 1 or 5 mg/kg cusatuzumab once in 21 days, and the regimen was generally tolerated, with adverse events predominantly mild to moderate.^57^ A total of 1, 5, and 9 cases of complete response (CR), partial response (PR), and SD were, respectively, documented, yielding an overall response rate of 23%.^57^ Notably, in patients with SS, the PR rate reached 50%, indicating a better efficacy compared to other subtypes, and this improvement was positively correlated with dose, increasing from 33% to 60% when cusatuzumab dose was elevated from 1 to 5 mg/kg.^57^ However, in a phase Ib trial involving 11 NPC cases, the efficacy of cusatuzumab was limited, with SD observed in only 2 patients, while the rest showed progressive disease (PD).^58^ Although both CTCL and NPC express CD70, the observed efficacy of cusatuzumab differs significantly. In CTCL, the therapeutic effect is likely mediated by direct tumour cytotoxicity via ADCC, leading to tumour cell apoptosis.^57^ In NPC, investigators have associated the limited efficacy with several possible reasons, including the small number of enrolled cases, the absence of CD70‐guided stratification and prior treatment‐related immunosuppression, which may have compromised the immune‐modulating efficacy of the antibody.^58^ Notably, the relative contributions of effector function (ADCC/CDC/ADCP) and the inhibition of CD70's interaction with CD27 (especially Tregs modulation) to the antitumour activity of cusatuzumab remain insufficiently defined and may vary across tumour types depending on immune contexture and tumour biology.^55^, ^56^, ^58^

In addition, Li et al.^59^ designed and synthesized another high‐affinity humanized antibody, IMM40H. Compared with cusatuzumab, IMM40H has a higher binding affinity for CD70 and stronger Fc‐mediated effector functions. In xenograft mouse models of multiple myeloma, Burkitt lymphoma, and RCC, .3 mg/kg IMM40H exhibited superior antitumour activity than 1.0 mg/kg cusatuzumab, suggesting enhanced therapeutic potential.^59^ Its clinical evaluation has entered Phase I (NCT05549557).

### 3.2 Antibody–drug conjugates

Since the use of cytotoxic drugs in cancer treatment, researchers have been seeking ways to improve efficacy while reducing overall toxicity to patients. ADCs, as an innovative therapeutic approach, have been developed based on monoclonal antibodies. ADCs covalently combine monoclonal antibodies and cytotoxic drugs (payloads) with linkers to, respectively, utilize their high specificity and high toxicity toward cancer cells.^60^ Due to the presence of the antibody, these drugs can locate CD70‐positive tumour cells, forming an ADC–antigen complex. This complex enters the cell through endocytosis and undergoes intralysosomal dissociation to release the payload.^61^ These cytotoxic drugs precisely kill tumour cells by inducing DNA damage, disrupting microtubules, and other mechanisms, while minimizing damage to normal tissues.^62^, ^63^

Several CD70‐targeted ADCs are currently in clinical or preclinical development. Nakae et al.^64^ developed a CD70‐ADC loaded with monomethyl auristatin F (MMAF) for treating uterine leiomyosarcoma via cytotoxic mechanisms. Shiomi et al.^65^ conjugated vorsetuzumab with MMAF to form a CD70‐ADC for the treatment of platinum‐resistant ovarian cancer. Although several ADCs showed promising results in early studies, some have been discontinued due to suboptimal efficacy or safety concerns.^66^ SGN‐75, developed by Seattle Genetics (now Seagen), is an ADC formed by conjugating a humanized IgG1 monoclonal antibody with MMAF via a maleimidocaproyl (mc) linker.^67^ Although SGN‐75 showed significant antitumour activity in pancreatic and ovarian cancer models, the first‐in‐human NCT01015911 study recruiting CD70‐positive metastatic RCC and non‐Hodgkin lymphoma (NHL) sufferers revealed that its clinical efficacy did not meet expectations. Among the 47 patients treated every 3 weeks, only 1 achieved CR and 2 achieved PR.^68^ Due to limited clinical efficacy and the emergence of dose‐limiting toxicities, Seagen ultimately discontinued the development of SGN‐75. The cytotoxic payload MMAF has been associated with ocular toxicity and thrombocytopenia, both of which were identified as key adverse events during the trial and contributed to the decision to terminate further development.^66^ MDX‐1203, another CD70‐targeted ADC comprising a fully human antibody and a duocarmycin‐derived cytotoxic payload, entered a phase I study (NCT00944905) involving 26 patients diagnosed with ccRCC or B‐cell NHL. Although 69% of patients achieved SD, no PR or CR were observed. Moreover, high‐dose administration (15 mg/kg) was associated with notable toxicities, including pleural and pericardial effusions, causing permanent therapy stoppage in 50% participants receiving this dose.^69^

However, the development of CD70‐targeted ADCs has not stalled, and the next‐generation CD70‐ADCs, such as SGN‐CD70A and AMG 172, have entered the research phase, aiming to achieve a better balance between efficacy and toxicity. SGN‐CD70A, also developed by Seagen, is a CD70‐targeted ADC with a pyrrolobenzodiazepine (PBD) dimer as its payload, a particularly effective DNA crosslinker. Compared with MMAF, the PBD dimer has a more direct mechanism of action and can exhibit higher toxicity at lower doses. Preclinical studies demonstrated that SGN‐CD70A suppressed proliferation and triggered apoptosis in CD70‐positive CTCL cells, with multiple high‐dose administrations significantly prolonging the survival of lymphoma mice.^70^ However, data from a phase I trial (NCT02216890) revealed concerns regarding both the therapeutic effectiveness and safety profile. In 20 CD70‐positive NHL patients, SGN‐CD70A showed some monotherapy activity, including 1 CR and 3 PR. However, up to 75% of patients developed frequent and severe thrombocytopenia.^71^ Similarly, in RCC, high‐dose treatment with SGN‐CD70A was also associated with severe toxicities, including anaemia and thrombocytopenia. Despite achieving a disease control rate of 78%, only one patient achieved PR, with the median progression‐free survival being as short as 3.5 months.^72^ These results restrict the clinical application potential of SGN‐CD70A. Similar to SGN‐CD70A, AMG 172, comprising a monoclonal antibody against human IgG1 linked by a noncleavable linker to maytansinoid as the payload, was evaluated by the NCT01497821 clinical study involving advanced ccRCC sufferers.^73^ Among 37 evaluable cases, only 5.4% achieved PR while 35.1% experienced PD, reflecting limited clinical activity despite manageable toxicity.^73^

Overall, it is challenging to develop ADCs targeting CD70 and apply them clinically.^66^ Their limited success to date is primarily due to insufficient antitumour activity in clinical settings and unacceptable toxicity profiles associated with current payloads and linker designs. The inability of preclinical models to accurately mimic human physiological complexity, particularly in toxicity prediction, further hinders translational success. Many ADCs that appear safe in animal studies may exhibit unanticipated adverse effects in clinical trials. Additional factors such as unstable linkers and nonspecific binding further narrow the therapeutic window. Therefore, future efforts should focus on optimizing ADC design, incorporating cleavable and tumour‐specific linkers, selecting better‐tolerated payloads, and exploring combination strategies or next‐generation platforms like bispecific ADCs or conditionally activated ADCs to improve therapeutic precision and reduce systemic toxicity.^61^, ^74^, ^75^

### 3.3 CAR‐T cell therapy

**FIGURE 3:**
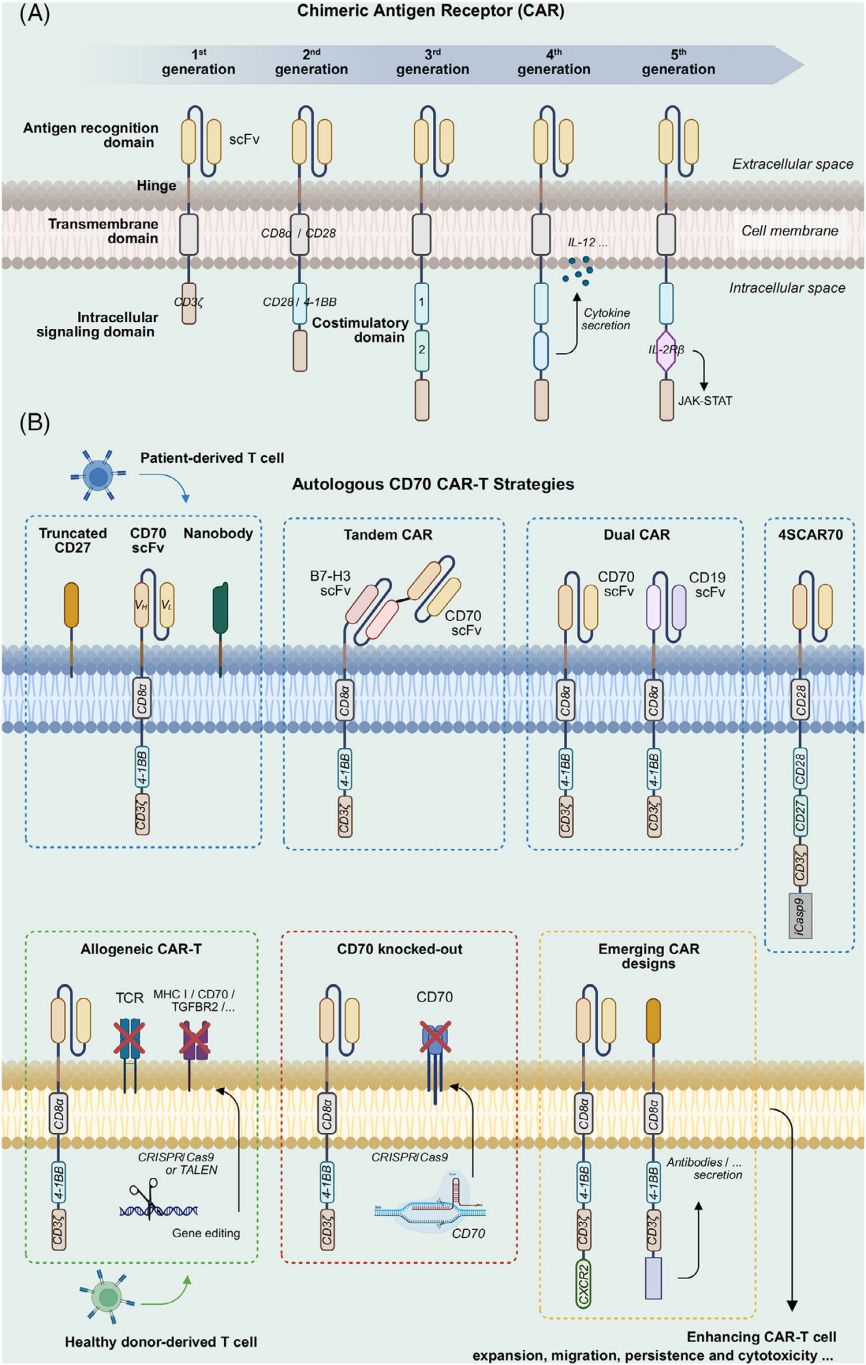
CD70‐targeted CAR designs and strategies. **A,** CAR construction across five generations. The first‐generation CAR consists of four key components: the antigen recognition domain (scFv), hinge, transmembrane domain, and intracellular signalling domain. The second generation introduces a costimulatory domain to enhance T cell activation. The third generation further expands upon the second generation by incorporating two or more costimulatory domains. The fourth‐generation CAR adds cytokine secretion elements to enhance the immune response within the tumour microenvironment. The fifth generation represents the latest design, which is more complex and employs multiple mechanisms to enhance antitumor activity and improve clinical safety. **B,** CD70‐targeted CAR‐T strategies. The first row (from left to right) illustrates autologous CD70 CAR‐T designs derived from patient T cells. These include three types of antigen recognition domains: truncated CD27, CD70 scFv, and nanobody, combined with appropriate costimulatory domains and hinge structures to enhance CAR‐T efficacy. The middle section features tandem CAR, which combines two antigen recognition domains targeting different tumour antigens within one receptor, and dual CAR, which features two distinct CAR molecules, each with an individual antigen recognition domain, allowing the CAR‐T cell to target multiple antigens simultaneously. The right section shows 4SCAR70, a fourth‐generation CAR with multiple co‐stimulatory domains and the iCasp9 suicide gene. The second row (from left to right) includes allogeneic CAR‐T cells derived from healthy donors, featuring TCR and immune‐modulatory gene editing to improve therapeutic potential. The CD70 knockout section shows the use of CRISPR/Cas9 technology to knockout endogenous CD70 in CAR‐T cells, enhancing production efficiency and cytotoxicity. The final section displays emerging CAR designs, which include modifications like CXCR2 for enhanced migration and the ability to secrete antibodies, cytokines, or other therapeutic molecules, aiming to optimize CAR‐T cell function.

As a type of adoptive immunotherapy, CAR‐T involves genetically modifying T cells to enable the expression of tumour antigen‐recognizing chimeric antigen receptors (CARs), thereby precisely targeting and eliminating tumour cells^76^ (Figure 3A). Identifying suitable new targets, optimizing existing CAR‐T designs, and developing novel CAR‐T therapies remain major research directions.^77^ The safety and efficacy of several CAR‐T strategies targeting CD70 have been assessed preclinically and within patient cohorts.^78^, ^79^ We summarize the common strategies for constructing CD70‐targeted CARs, including nanobody‐based CAR‐T, dual‐target CAR‐T, and allogeneic CAR‐T, which will be discussed in detail later (Figure 3B).

#### 3.3.1 Research progress of autologous CD70‐targeted CAR‐T cells

Autologous CAR‐T cells come from a patient who sequentially undergoes genetic modification, ex vivo expansion, and reinfusion, providing benefits such as minimal immune rejection risk and sustained efficacy. Among CD70‐targeted CAR‐T approaches, autologous CAR‐T represents the most widely utilized one. However, existing evidence reflects that CAR‐T cells targeting CD70 may display insufficient expansion capacity and/or limited persistence in vivo, which could contribute to suboptimal treatment outcomes in some cases.^80^ Selecting appropriate extracellular antigen recognition and co‐stimulatory domains, as well as optimizing hinge designs, can help enhance functional persistence and in vivo efficacy (Figure 3B). Common extracellular domains include truncated CD27 (trCD27) and CD70‐specific single‐chain variable fragment (scFv). CAR‐T cells designed based on both of these domains have shown good antitumour activity in glioma mouse models.^32^, ^38^ Preclinical study has shown that CAR‐T cells with heavy–light chain (H/L) scFv configurations outperform those with light–heavy chain (L/H) in transduction efficiency and proliferation. While trCD27 CAR‐T cells exhibited superior expansion capabilities in CD19‐negative B‐cell lymphoma (BCL) models preclinically, their functional performance remained unconfirmed in the study.^81^ By comparing different CAR design strategies, it was demonstrated that trCD27‐41BB‐zeta CAR‐T cells exhibited the strongest IFN‐γ production and were capable of eradicating CD70‐positive tumours in mice.^82^ Tim et al.^83^ demonstrated that CD27z‐CAR T cells, connecting CD27 to CD3‐zeta, showed reduced T‐cell differentiation tendencies, enhanced expansion, and improved persistence in the acute myeloid leukaemia (AML) model. The study also found that CD70 scFv‐based CARs incorporating a long‐flexible hinge and 4‐1BB co‐stimulatory motif exhibited enhanced antitumour responses and improved immune memory.^83^ Nevertheless, some studies indicated that CD70 scFv outperforms trCD27 in affinity and cytotoxicity across multiple solid tumour models.^84^

In recent years, a novel extracellular domain known as the nanobody has become a research focus in CAR‐T therapy because of its distinct advantages.^85^ Nanobodies (VHHs) are antibody fragments that are produced based on the camelid heavy‐chain‐only immunoglobulin variable region.^86^ Compared with traditional scFv, nanobodies have the advantages of smaller molecular weight and lower immunogenicity. Their single‐domain structure exhibits stronger resistance to extreme conditions, including low pH and high temperatures, enhancing CAR‐T cell stability, better adapting to complex tumour microenvironments, and reducing potential immune‐related side effects.^87^, ^88^ Despite their modularity and favourable biophysical properties, nanobody‐based CAR‐T cells may face limitations related to rapid renal clearance, potential immunogenicity, manufacturing scalability, and overall production cost.^89^, ^90^, ^91^ In preclinical RCC animal and cell models, CD70 CAR‐T cells incorporating nanobodies demonstrated enhanced cytokine production and greater antitumour efficacy relative to scFv‐based CAR‐T cells,^92^ suggesting that nanobody‐based CAR constructs hold potential for improving CD70‐directed CAR‐T therapies against solid tumours and merit further clinical evaluation.^92^

The heterogeneity of CD70 expression across tumour cells also evidently undermines the efficacy of CD70‐specific CAR‐T cell therapy.^32^ For instance, in AML, CD70 is present on AML blasts, leukaemia stem cells (LSCs), and leukemic progenitor cells, but its expression exhibits considerable inter‐patient and intra‐sample variability.^34^, ^80^ Preclinical evidence demonstrated that conventional CAR‐T cells showcased diminished toxicity toward AML cells with low CD70 expression, highlighting the impact of antigen density on therapeutic response.^80^ Using CRISPR/Cas9 editing, T cells were modified to express an anti‐CD70 scFv at the TRAC site, yielding HLA‐independent TCR‐engineered (HIT‐T) cells.^93^ These HIT‐T cells exhibited greater antigen sensitivity and enhanced degranulation compared to conventional CAR‐T cells, thereby improving therapeutic efficacy for tumour cells with low CD70 expression and effective tumour growth suppression in preclinical models.^93^ Silva et al.^94^ incorporated a function into conventional CAR‐T cells that allows the secretion of a CD33‐targeted T cell‐engaging antibody molecule by the cells. By co‐targeting CD70 and CD33, they aimed to address antigen heterogeneity in AML treatment and mitigate systemic toxicity in a preclinical model, while simultaneously redirecting untransduced bystander T cells to CD33‐positive tumour cells to enhance antitumour activity.^94^ TanCAR‐T cells that can simultaneously target B7‐H3 and CD70 were fabricated via a tandem scFv design.^84^ These dual‐specific CAR‐T cells showcased superior anticancer efficacy relative to single‐target counterparts in lung cancer and melanoma models.^84^ These innovative designs provide potential strategies to mitigate antigen escape, though further validation is required in clinical settings.

Several studies have indicated that patients with hematologic malignancies treated with CAR‐T cells targeting CD19 may undergo disease recurrence due to antigen loss.^81^, ^95^ In this context, CD70 is an emerging alternative target in managing CD19‐negative BCL. A clinical trial (NCT03125577) documented one participant suffering from refractory, recurrent primary central nervous system DLBCL that achieved sustained remission following administration of fourth‐generation CAR‐T cells targeting both CD19 and CD70.^96^ The fourth‐generation CAR‐T cells (4SCAR) used in this study consisted of CD19/CD70‐specific scFvs, multiple signalling domains including CD28, CD27, and CD3ζ, as well as the inducible suicide gene iCasp9^95^, ^96^ (Figure 3B). In the 17‐month follow‐up, the patient showed no disease relapse and did not experience severe adverse events, involving CAR‐T‐related encephalopathy syndrome (CRES) or cytokine release syndrome (CRS), suggesting a potentially favourable safety profile.^96^ These promising findings need to be clinically validated utilizing additional patient cohorts to ascertain the safety benefits of suicide gene integration.

#### 3.3.2 Innovation advances in allogeneic CD70‐targeted CAR‐T cells

Despite achieving promising disease control rates in some studies, autologous CD70‐targeted CAR‐T cells face several limitations. The highly personalized manufacturing model results in long production cycles and high costs, and the efficacy may be compromised by unsatisfactory patient‐derived T cell quality.^39^, ^97^ In contrast, allogeneic CAR‐T cells can be obtained in bulk from healthy donors and follow an “off‐the‐shelf” model, overcoming individual variability and production cycle limitations.^98^, ^99^ They are expected to offer greater scalability and therapeutic potential, which may help address existing difficulties faced by the CAR‐T strategy.

ALLO‐316 and CTX130/CTX131 are two representative allogeneic CAR‐T constructs targeting CD70, developed using TALEN and CRISPR/Cas9 gene‐editing technologies, respectively, offering different design approaches and optimization strategies^100^, ^101^ (Figure 3B). The FDA has recently granted regenerative medicine advanced therapy designation for ALLO‐316 in managing CD70‐positive advanced or metastatic RCC. Utilizing TALEN technology, it achieves TCR and CD52 gene knockout, thereby preventing graft‐versus‐host disease (GvHD) and enhancing chemotherapy tolerance.^100^ CTX130, on the other hand, involves CD70 knockout and targeted integration of the CAR construct into the TRAC locus of T cells, enabling efficient and stable CAR expression while eliminating TCR to prevent GvHD.^101^ In the COBALT‐RCC phase I clinical trial (NCT04438083), CTX130 achieved a disease control rate of 81.3%, with one patient experiencing CR and showing no disease progression after 3 years.^101^ This result highlights the potential of CTX130 in solid tumours. In the recently released COBALT‐LYM phase I clinical trial (NCT04502446), CTX130 demonstrated manageable safety and encouraging anticancer efficacy among participants suffering from relapsed or refractory T‐cell lymphoma. The objective response rate at dose level 3 and above reached 51.6%, with 19.4% achieving CR.^102^ However, CTX130 has limitations, including insufficient persistence in vivo (shorter than autologous CAR‐T cells) and a relatively higher incidence of CRS. The research team developed CTX131 to improve the durability and potency of CAR‐T cells through knocking down Regnase‐1 and TGFβR2 on the basis of CTX130, which is also now being assessed by a phase I/II clinical study (NCT05795595).^101^

Recently, Zhang et al.^103^ introduced an innovative strategy aimed at addressing limitations of allogeneic CAR‐T cell therapy. The researchers constructed a universal CD70 CAR‐T product (SAP UCAR‐T) with three genes (HLA‐DRA, B2M, and TRAC) knocked out to reduce host‐versus‐graft immune responses.^103^ Furthermore, they incorporated a synthetic SAP (self‐activating and protecting) module consisting of the CD47 extracellular domain, a mutated IL‐7Rα transmembrane domain, and its intracellular signalling domain.^103^ This fusion construct not only protected CAR‐T cells from being scavenged by host macrophages and NK cells but also activated IL‐7 signalling pathways to enhance their proliferation and persistence.^103^ These features support its potential to facilitate allogeneic CD70 CAR‐T therapy application for the management of solid malignancies.

#### 3.3.3 CD70 knockout: A key to optimizing CAR‐T cell manufacturing and efficacy?

In fact, CD70 is not a tumour‐specific antigen. Several studies have shown that its surface expression on T cells, while acting as a target antigen, may limit CAR‐T cells’ productivity and functional activity through multiple mechanisms. First, CAR‐T cells recognizing the target antigen on the surface of T cells may trigger fratricide, leading to failed expansion, reduced activity, or even T‐cell apoptosis.^97^, ^104^ Although certain scFv‐constructed CAR structures avoid CD70 recognition and fratricide through a “cis‐masking” mechanism, this approach is still imperfect and may fail under different CAR designs or manufacturing conditions.^81^, ^100^ In contrast to fratricide, cis‐interaction involves the binding of CD70 to CAR molecules on the same CAR‐T cell surface. This “self‐binding” does not induce intercellular attacks or apoptosis but triggers exhaustion‐related signalling cascades in CAR‐T cells, adversely affecting T‐cell status during in vitro expansion.^105^ Second, abnormal interactions between CD70 and its ligand CD27 may cause CAR‐T cell overactivation and early exhaustion, triggering negative feedback mechanisms that suppress T‐cell proliferation and trigger apoptosis.^106^ As an inhibitory receptor, CD70 expression may further weaken CAR‐T cells’ anticancer capability, limiting their therapeutic potential.^107^ Therefore, it can be inferred that the expression of endogenous CD70 significantly impacts CAR‐T cell manufacturing and functionality. Using gene‐editing technologies like CRISPR/Cas9 to knockout CD70 can effectively address these issues, improving CAR‐T cell manufacturing efficiency and quality while increasing the percentages of central memory and naïve T cells, thereby achieving more durable antitumour effects^105^, ^108^ (Figure 3B). However, it should be emphasized that CD70 knockout does not universally enhance CAR‐T cell performance. One study found that TALEN‐mediated CD70 deletion had no appreciable impact on the proliferative potential or cytotoxicity of CAR‐T cells in vitro, and showed limited efficacy in RCC models with low CD70 expression.^100^

Despite many advances in optimizing durability and efficacy with multitargeted, universal, and other CAR‐T design strategies, the satisfactory efficacy of CAR‐T therapies toward haematological malignancies has not fully carried over to the solid tumour arena. This is primarily due to the complex immunosuppressive microenvironment, antigen heterogeneity, and the challenges CAR‐T cells encounter in effectively infiltrating deep into solid tumours.^109^, ^110^, ^111^ As previously described, multiple schemes are being explored to address these issues, including nanobody‐based CAR designs,^92^ TGFβR2 knockout to mitigate suppressive signalling,^101^ and incorporation of synthetic SAP modules to reduce host immune rejection.^103^ In addition, the integration of IL‐8 receptors into CAR‐T cells has shown potential to promote trafficking toward tumour sites.^112^ While these approaches have demonstrated encouraging preclinical results, their clinical efficacy and safety remain to be validated in ongoing and future studies. Moreover, CD70‐targeted CAR‐T therapies exhibit toxicity profiles similar to other CAR‐T platforms, including CRS and neurotoxicity, which may hinder broader clinical implementation and require vigilant management.^102^, ^113^ These limitations in efficacy, safety, and accessibility highlight the need to refine CAR‐T product formulation and delivery approaches, and to investigate alternative immune cell‐based therapies, including CAR‐NK and macrophage‐derived CAR strategies.^114^

### 3.4 CAR‐NK cell therapy

NK cells possess a unique target recognition mechanism that differs from T cells, allowing them to exert innate cytotoxicity against tumour cells. When engineered with CARs, CAR‐NK cells gain enhanced specificity and retain their natural antitumour properties. Since NK cells lack TCR expression, CAR‐NK cells have a minimal risk of inducing GvHD and exhibit a low incidence of serious toxicities such as CRS or neurotoxicity.^115^ More importantly, they can be sourced from allogeneic donors, overcoming the limitations of autologous cell therapy, expanding cell sources, and significantly reducing treatment costs.^116^

**FIGURE 4:**
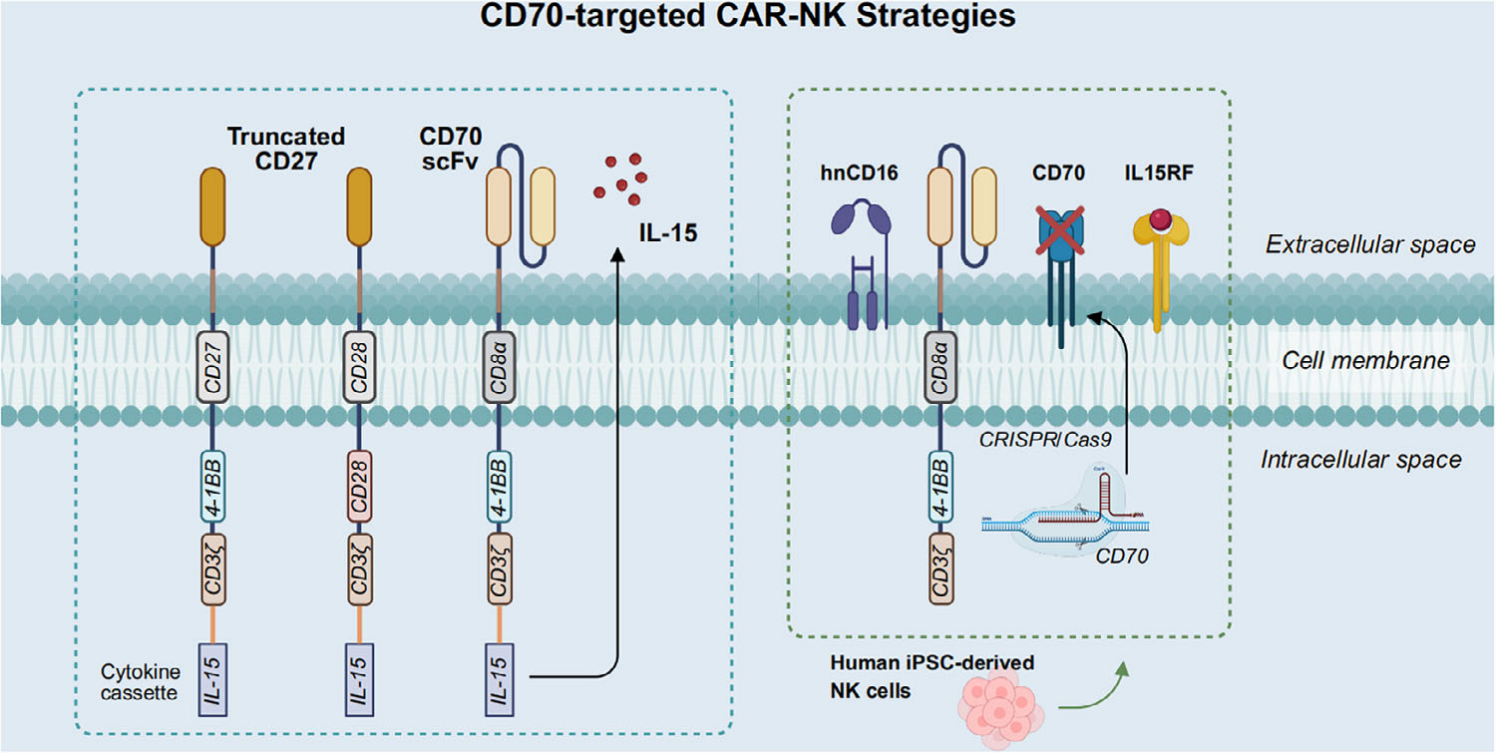
CD70‐targeted CAR‐NK strategies. The left section illustrates current fourth‐generation CD70 CAR‐NK designs that include various antigen recognition domains, transmembrane domains, and co‐stimulatory domains, integrated with IL‐15 secretion modules to enhance NK cell persistence and cytotoxicity. The right section illustrates a multiengineered CD70 CAR‐NK cell strategy derived from human induced pluripotent stem cell (iPSC), incorporating CD70 knockout to prevent fratricide, high‐affinity noncleavable CD16 (hnCD16) to augment antibody‐dependent cellular cytotoxicity, and an IL‐15Rα/IL‐15 fusion protein (IL15RF) to enhance persistence. This schematic was redrawn and conceptually adapted from the CAR‐NK strategy developed by Wang et al.,^115^ and reorganized to highlight its key engineering elements.

CD70‐targeted CAR‐NK cells remain in early‐stage research, yet current findings show promising application potential. The incorporation of cytokines, including IL‐15, has been reported to evidently enhance their persistence and antitumour function^117^ (Figure 4). Eynde et al.^118^ designed CD70‐CAR‐IL‐15 NK cells to target CD70‐positive colorectal and pancreatic ductal adenocarcinoma cells as well as cancer‐associated fibroblasts (CAFs). That research exhibited that IL‐15 enhanced the cytotoxicity of CAR‐NK cells through promoting CAR expression and elevating proinflammatory cytokine secretion.^118^ Additionally, IL‐15 can form an autocrine positive feedback loop that further activates and enhances the antitumour capacity of NK cells.^118^ Another study demonstrated in CD19‐negative BCL that repeated dosing strategies, by continuously increasing IL‐15 levels in vivo, further enhanced the persistence and proliferation of CD70‐CAR NK cells, thereby promoting their antitumour effects.^37^ Wang et al.^119^ developed human induced pluripotent stem cell (iPSC)‐derived 70CAR‐iNK cells that incorporated IL‐15 receptor α/IL‐15 fusion protein (IL15RF), high‐affinity noncleavable CD16, and CD70 gene knockout, significantly enhancing NK cell cytotoxicity and persistence (Figure 4). These cells also reduced allogeneic T‐cell immune responses, avoiding immune rejection issues commonly seen in traditional cell therapies.^119^

In a preclinical study, Acharya et al.^120^ investigated the impact of different co‐stimulatory molecules on CAR‐NK cell activity. They generated CD70‐targeted CAR constructs by fusing the extracellular domain of CD27 with cytoplasmic domains from multiple co‐stimulatory molecules. Despite the lack of endogenous CD28 expression in NK cells, CAR27‐28ζ NK cells featuring CD28 co‐stimulation exhibited superior long‐term cytotoxicity and multifunctionality^120^ (Figure 4). Corresponding phase I/II clinical trials are currently underway to assess the safety and efficacy of CAR27‐28ζ NK cells in patients with CD70‐positive hematologic and solid malignancies (NCT05092451, NCT05703854).

Despite encouraging progress, the clinical translation of CAR‐NK therapies remains limited by several challenges, including poor gene transfer efficiency, limited in vivo persistence, and suboptimal trafficking to solid tumours.^121^, ^122^ Strategies such as IL‐15 armouring, inhibitory receptor knockout, and metabolic reprogramming have shown promise, but improvements in manufacturing scalability and functional durability are still needed to enable broader clinical application.^115^, ^123^

### 3.5 Combination therapy strategies

Although each of these therapies has made significant progress in its respective fields, the limitations of monotherapy remain prominent. Heterogeneity of tumour cells, development of drug resistance, immune escape, and loss or alteration of therapeutic targets often limit the durability and efficacy of therapy.^124^ In addition, the systemic toxicity of certain therapies cannot be ignored, which further narrows the patient's treatment options. In this context, combination therapy strategies have become the optimal solution for antitumour therapy.^125^, ^126^

Current combination therapy strategies targeting CD70 typically integrate traditional therapies with emerging modalities such as monoclonal antibodies or CAR‐T/CAR‐NK cell therapies. These combinations have been investigated in preclinical models and have shown potential to augment antitumour responses through multiple mechanisms, including upregulating CD70 antigen expression, enhancing the cytotoxicity of CD70‐targeted immune cells, and remodelling the immunosuppressive tumour microenvironment to overcome resistance.^127^

In AML, research has found that hypomethylating agents (HMAs) can upregulate CD70 expression in LSCs by reducing CD70 promoter methylation and enhance stem cell self‐renewal and survival by activating the CD70/CD27 signalling pathway.^128^ Preclinical studies have further shown that decitabine‐induced CD70 upregulation sensitizes AML LSCs to cusatuzumab, with the combination leading to a marked reduction of functional LSCs in vitro and in vivo. Quantitative analysis using the Chou–Talalay method confirmed a synergistic interaction between the two agents.^129^ Based on this mechanism, several clinical trials have evaluated the combination of cusatuzumab with azacitidine. A phase I/II clinical trial (NCT03030612) demonstrated promising activity, with 8 of 12 AML patients achieving CR, 2 complete remissions with incomplete blood count recovery, and 2 PR.^129^ Although the trial was not designed as a comparative study, the observed response rate was described as favourable in relation to historical data on HMA monotherapy.^129^ A subsequent phase I/II trial refined the dosing schedule of cusatuzumab (1, 3, 10, and 20 mg/kg) in combination with azacitidine, establishing 10 mg/kg as the recommended phase II dose.^130^ Of the 38 participants enrolled 19 exhibited objective responses, including 14 with CR, supporting the therapeutic potential of this combinatorial approach.^130^ In the CULMINATE randomized phase II clinical trial (NCT04023526), researchers compared the efficacy of two doses of cusatuzumab (10 vs. 20 mg/kg) combined with azacitidine. The results demonstrated that the CR rate (27%) in the 20 mg/kg group was significantly higher than in the 10 mg/kg group (12%), with superior overall efficacy.^131^ Besides, in another study involving six Japanese AML patients (NCT04241549), the combination therapy was found to be safe, but its efficacy was relatively limited, with no CR observed.^132^

Moreover, Cheng et al.^108^ found that the epigenetic modulators decitabine and chidamide were able to upregulate CD70 expression in AML cells, which in turn enhanced the targeted killing effect of nanobody‐based nb70CAR‐T cells. However, the precise regulatory mechanism of epigenetic modulators in promoting CAR‐T therapy and their in vivo efficacy still needs further validation. Similarly, in chronic myeloid leukaemia, imatinib, a tyrosine kinase inhibitor (TKI), was found to upregulate CD70 expression on LSCs via epigenetic mechanisms, and combining anti‐CD70 antibodies with TKIs effectively eliminated resistant LSCs by disrupting compensatory CD70/CD27‐mediated Wnt signalling.^133^ In solid tumours, a preclinical study has shown that low‐dose chemotherapy agents such as docetaxel and cisplatin can further upregulate the expression of CD70 in NSCLC. Sequential treatment combining chemotherapy and cusatuzumab can enhance the antitumour activity of NK and T cells, thereby inhibiting tumour growth.^134^

In addition to upregulating CD70 expression, multiple preclinical combination strategies have been investigated to enhance the efficacy of CAR‐T/CAR‐NK cells. These strategies act through different mechanisms, including boosting CAR cell persistence and activity, altering tumour cell susceptibility, and modulating the immunosuppressive microenvironment. Leick et al.^136^ reported that azacitidine upregulated CD70 expression in AML cells and facilitated the expansion of CD8H&TM CD70 CAR‐T cells modified with CD8 hinge and transmembrane domains, thereby enhancing antitumour efficacy.^135^ Activation of the IL‐21/IL‐21R signalling pathway has been implicated in enhancing the sensitivity of LSCs to CD70 CAR‐T cell therapy and cytarabine by modulating their differentiation and metabolic state, thereby contributing to enhanced therapeutic efficacy in combination settings.^137^ Proteasome inhibitors such as bortezomib and carfilzomib have been shown to enhance the cytotoxic activity of CAR‐NK cells against AML by downregulating HLA‐I molecules expression and upregulating stress‐associated proteins.^138^ In RCC, Ji et al.^139^ reported that PARP inhibitors enhanced the efficacy of CD70‐targeted CAR‐T therapy via activation of the cGAS‐STING signalling cascade in tumour cells, resulting in elevated CXCL10 and CCL5 expression and promoting CAR‐T cell infiltration and cytotoxicity. Additionally, in glioblastoma, oncolytic herpes simplex virus‐1 (oHSV‐1) was shown to synergize with CD70 CAR‐T cells by promoting IFN‐γ release, suppressing TGF‐β1 expression, and modulating Tregs infiltration, thereby remodelling the immunosuppressive microenvironment and enhancing treatment responses.^140^

CD70‐targeted therapies have also been investigated in combination with ICIs. CD70 has been found to correlate with immune checkpoint gene expression across various cancers.^25^, ^26^ In pleural mesothelioma, co‐expression of CD70 and PD‐L1 was associated with poor prognosis and increased infiltration of FOXP3⁺ Tregs and CD163⁺ macrophages, supporting their cooperative role in immune evasion.^141^ In thyroid cancer, Ning et al.^142^ identified a mechanism of PD‐1 resistance in which M2 macrophage‐derived extracellular vesicles delivered miR‐21‐5p to suppress METTL3 expression, resulting in the demethylation and stabilization of CD70 mRNA. This upregulation of CD70 promoted Tregs accumulation and T cell exhaustion, contributing to an immunosuppressive tumour microenvironment. Blockade of CD70 with cusatuzumab reversed these effects and partially restored sensitivity to PD‐1 inhibition.^142^ In NPC, a combination of cusatuzumab and the anti‐PD‐1 antibody nivolumab demonstrated superior tumour control compared with either monotherapy in patient‐derived organoids and humanized mouse models.^29^

These combination strategies, involving CD70‐targeted therapies with chemotherapy, targeted agents, ICIs and other adjunctive approaches, have shown promise in remodelling the immunosuppressive tumour microenvironment and enhancing tumour‐specific immune responses. While these strategies show therapeutic potential, most remain at the experimental stage, and clinical data remain limited. Furthermore, whether these combinations offer superior clinical efficacy over monotherapy has yet to be confirmed, and synergistic effects have not been systematically evaluated in most studies. These limitations underscore the need for mechanistic investigations and well‐designed clinical trials to determine their true therapeutic value.

## 4 DISCUSSION

While CD70 has been extensively studied in hematologic and solid malignancies, growing evidence suggests that it also exerts important immunomodulatory functions in nontumour settings.^143^, ^144^ In various autoimmune disorders, including alopecia areata, systemic lupus erythematosus, and multiple sclerosis, elevated CD70 expression has been linked to disease severity and activity, and the overactivation of the CD70‐CD27 signalling pathway exacerbates the autoimmune response.^145^, ^146^, ^147^ Moreover, CD70 has been proposed as a biomarker of immune reconstitution in HIV‐infected individuals, with elevated CD70 expression on CD4+ T cells contributing to immune activation and HIV‐associated inflammation.^148^ Studies have also shown that CD70 plays a regulatory role in the maintenance of redox balance and vascular function in endothelial cells and promotes inflammation in acute GvHD.^149^, ^150^ Therefore, CD70 holds significant clinical value not only in the diagnosis and treatment of CD70‐positive tumours but also as a potential breakthrough for the treatment of immune‐related diseases.

Molecular imaging with ^89^Zr‐Atezolizumab PET has shown value in noninvasive evaluation of PD‐L1, aiding in the assessment of expression heterogeneity, anticipating immunotherapeutic responses, and supporting patient stratification for treatment.^151^, ^152^, ^153^ These studies provide insights into CD70‐targeted imaging. CD70‐targeted immuno‐PET/CT, as a novel noninvasive functional molecular imaging technique, has potential applications in RCC and other CD70‐high‐expressing tumours, including precise detection of early micrometastases, monitoring postoperative recurrence and metastasis, screening patients suitable for CD70‐targeted therapy and optimizing individualized treatment strategies, dynamic monitoring of disease progression and efficacy evaluation, and guiding the development of targeted drugs.^154^ Co‐imaging of CD70 with other tumour markers (e.g., CAIX) could also be explored in the future for a more comprehensive assessment of the tumour microenvironment and disease state.^28^ In addition, by combining radionuclides such as ^177^Lu or ^225^Ac with CD70‐specific antibodies, radioimmunotherapy can be further developed to achieve precise targeted therapy.^28^, ^155^ However, there is a lack of clear clinical evidence demonstrating a significant correlation between patient CD70 expression levels and the therapeutic efficacy of CD70‐targeted therapies.^46^, ^102^ More large‐scale clinical studies are still needed to comprehensively assess the predictors of the effectiveness of CD70‐targeted therapies.

Given its close association with tumour immune regulation, CD70‐targeted therapies not only enable direct elimination of CD70‐positive tumour cells but also hold potential to activate effector immune responses and remodel the immunosuppressive microenvironment. We systematically summarize the latest research progress in CD70‐targeted therapies (Table 2). Despite promising progress, important limitations and challenges remain. Clinical outcomes vary markedly across different tumour types, with CD70‐targeted strategies generally achieving more favourable responses in hematologic malignancies compared with solid tumours. In solid tumours, factors such as the immunosuppressive microenvironment and poor infiltration of effector cells collectively impair therapeutic efficacy.^109^, ^121^ Moreover, monoclonal antibodies and ADCs often induce hematologic toxicities, including thrombocytopenia, anaemia, and neutropenia. In CAR‐T‐cell therapies, endogenous CD70 expression on T cells may cause fratricide during manufacturing, potentially impairing CAR‐T expansion and functionality. CRS, neurotoxicity, and other treatment‐related adverse events also represent barriers to broader clinical application. Although CD70 is normally restricted in expression, its upregulation on activated immune cells may theoretically pose a risk of on‐target, off‐tumour toxicity during therapeutic targeting.^26^, ^156^ While such adverse effects have not been definitively observed in current preclinical or clinical studies, lessons from CAR‐T cell therapies against other antigens (e.g., CAIX, HER2, EGFR) highlight the potential for severe or irreversible off‐tumour toxicity when targeting antigens with low‐level normal tissue expression, underscoring the need for careful safety evaluation and long‐term monitoring.^157^

**TABLE 2:** CD70‐targeted therapies in clinical trials.

Name	Cancer	Clinical trial identifier	Phase	Status	PMID
**Monoclonal antibodies**					
Cusatuzumab (ARGX‐110)	Advanced CD70+ malignancies	EudraCT 2012‐005046‐38	Phase I	Completed	28765328
	CTCL	NCT01813539	Phase I/II	Completed	34726773
	NPC	NCT02759250	Phase I	Completed	34405542
MDX‐1411	ccRCC	NCT00656734	Phase I	Completed	
IMM40H	Advanced CD70+ malignancies	NCT05549557	Phase I	Unknown	37849799
SEA‐CD70	MDS, AML	NCT04227847 EudraCT 2019‐001917‐18	Phase I	Recruiting	
ADCs					
SGN‐75	NHL, RCC	NCT01015911	Phase I	Completed	25142258
MDX‐1203	NHL, RCC	NCT00944905	Phase I	Completed	26576779
SGN‐CD70A	NHL, RCC	NCT02216890	Phase I	Completed	30132271 30624766
AMG 172	ccRCC	NCT01497821	Phase I	Completed	30915497
PRO1160	RCC, NPC, NHL	NCT05721222	Phase I/II	Recruiting	
ARX305	Advanced CD70+ malignancies	CTR20222161	Phase I	Recruiting	
**CAR‐T cell**					
4SCAR19/20/22/30/38/70/123	BCL	NCT03125577	Phase I/II	Unknown	33727525 31867275
Anti‐hCD70 CAR‐transduced peripheral blood lymphocytes	Advanced CD70+ malignancies	NCT02830724	Phase I/II	Recruiting	27803044
CD70 CAR‐T cells	RCC	NCT05420519	Phase I	Recruiting	
	AML, NHL, MM	NCT04662294	Phase I	Recruiting	
	Solid tumors	NCT05420545	Phase I	Recruiting	
	Solid tumors	NCT05468190	Phase I	Recruiting	
	Solid tumors	NCT05518253	Phase I	Recruiting	
8R‐70CAR T cells	GBM	NCT05353530	Phase I	Recruiting	
Bi‐4SCAR CD19/70 T cells	B cell malignancies	NCT05436496	Phase I/II	Recruiting	
Bi‐4SCAR PSMA/CD70 T cells	CD70+ and/or PSMA+ malignancies	NCT05437341	Phase I/II	Recruiting	
Bi‐4SCAR GD2/CD70 T cells	CD70+ and/or GD2+ malignancies	NCT05438368	Phase I/II	Recruiting	
ALLO‐316	ccRCC	NCT04696731	Phase I	Recruiting	35294525
CTX130	RCC	NCT04438083	Phase I	Terminated	38583184
	T or B cell malignancies	NCT04502446 EudraCT 2019‐004526‐25	Phase I	Terminated	39617017
CTX131	Solid tumors	NCT05795595	Phase I/II	Recruiting	38583184
CAR‐NK cell					
CAR.70/IL15‐transduced CB‐NK cells	AML, BCL, MDS	NCT05092451	Phase I/II	Recruiting	38900051
	RCC, mesothelioma, osteosarcoma	NCT05703854	Phase I/II	Recruiting	38900051
DualCAR‐NK19/70 cell	NHL	NCT05842707	Phase I/II	Recruiting	
CB dualCAR‐NK19/70	NHL	NCT05667155	Phase I	Recruiting	
Combination therapy					
Cusatuzumab + azacitidine	MDS, AML	NCT03030612 EudraCT 2016‐002151‐17	Phase I/II	Completed	32601337 36779592
	AML	NCT04241549	Phase I	Completed	36394119
	AML	NCT04023526 EudraCT 2019‐000473‐23	Phase II	Active	37914483
Cusatuzumab + radiotherapy and/or chemotherapy	NPC	NCT02759250	Phase I	Completed	34405542
SGN‐75 + everolimus	RCC	NCT01677390	Phase I	Terminated	
SEA‐CD70 + azacitidine	MDS, AML	NCT04227847 EudraCT 2019‐001917‐18	Phase I	Recruiting	
Cusatuzumab + venetoclax + azacitidine	AML	NCT04150887 EudraCT 2019‐002808‐41	Phase I	Active	
Others					
MP0533	AML, MDS	NCT05673057 EudraCT 2022‐002432‐31	Phase I/II	Recruiting	38683145

Abbreviations: ADC, antibody‐drug conjugate; AML, acute myeloid leukaemia; BCL, B‐cell lymphoma; CAR‐NK, chimeric antigen receptor natural killer cell; CAR‐T, chimeric antigen receptor T cell; ccRCC, clear cell renal cell carcinoma; CD, cluster of differentiation; CTCL, cutaneous T‐cell lymphoma; CTR, Clinical Trials Register; EudraCT, European Union Drug Regulating Authorities Clinical Trials; GBM, glioblastoma.; MDS, myelodysplastic syndrome; MM, multiple myeloma; NCT, National Clinical Trial; NHL, non‐Hodgkin lymphoma; NPC, nasopharyngeal carcinoma; RCC, renal cell carcinoma.

Novel combination therapies and engineering strategies are under active investigation to address these challenges, as summarized in Table 2. Additionally, emerging therapies further enrich CD70‐targeted treatment strategies, including CD70‐specific fusion nanobody Nb3B6‐C3Fab that recruits endogenous IgG^158^; multispecific CD3‐engaging designed ankyrin repeat protein (DARPin) MP0533,^159^ which efficiently recognizes multiple targets and achieves precise killing by recruiting and activating T cells; and CD70‐based multiepitope vaccines.^160^ Notably, CD70‐targeted therapies hold promise as adjuncts to current therapeutic regimens, particularly in CD70‐positive tumours with limited response or acquired resistance to chemotherapy, immunotherapy, or targeted therapies. Their integration—as sensitizing agents or immune enhancers—may enhance treatment depth and durability. Nevertheless, most supporting evidence to date remains preclinical or derived from early‐phase trials, and further studies are needed to define optimal timing, combination regimens, and patient selection criteria. Future research should further optimize the design of targeted drugs and engineered immune cells to balance efficacy, durability, and toxicity, while continuing to explore combination therapies such as cusatuzumab with venetoclax and azacitidine (NCT04150887), which aim to eliminate tumour cells through complementary mechanisms of action and may enhance therapeutic efficacy by overcoming resistance.

CD70, as a pan‐cancer target abnormally overexpressed across various hematologic and solid tumours while maintaining highly restricted expression in normal tissues, demonstrates significant clinical potential in diagnosis, treatment, and prognostic assessment.^161^ Despite these advances, recent studies have revealed that CD70 is aberrantly expressed not only in tumour cells but also in specific stromal components within the tumour microenvironment.^26^ In colorectal cancer, a distinct subset of CD70‐positive CAFs has been identified, which promotes tumour progression by enhancing tumour cell migration and facilitating immune evasion through the accumulation of Tregs.^162^, ^163^ Similarly, elevated CD70 expression has been observed in CAFs and endothelial cells within head and neck squamous cell carcinoma, although the functional consequences remain insufficiently explored.^164^ These findings highlight the emerging complexity of CD70 biology beyond tumour cells themselves. The specific roles of these CD70‐positive nontumour cells in tumour progression, metastasis, and therapeutic resistance remain poorly defined. Future research should further investigate the regulatory mechanisms and functional roles of CD70 in diverse stromal and immune cell populations across different tumour microenvironments, thereby providing a more robust foundation for the optimization of CD70‐targeted strategies. In conclusion, CD70 represents a compelling molecular target with potential for integrated diagnostic and therapeutic applications across multiple tumour types. This approach may also inspire the development of similar strategies for other tumour markers, providing new directions for personalized and precision oncology.

## 5 CONCLUSION

CD70 is aberrantly overexpressed in multiple hematologic and solid tumours while exhibiting minimal expression in normal tissues, making it an attractive target for integrated cancer diagnosis and therapy. CD70‐targeted immuno‐PET/CT has demonstrated early promise in detecting both primary and metastatic RCC lesions and may further support patient stratification and therapeutic monitoring. Monoclonal antibodies, ADCs, CAR‐T, and CAR‐NK therapies have demonstrated encouraging preclinical activity and early clinical signals in select malignancies. While barriers such as immune‐related toxicity and tumour heterogeneity remain, innovative cell engineering and combination strategies continue to expand therapeutic possibilities. Notably, the integration of CD70‐targeted imaging with therapeutic strategies remains underexplored and represents a promising avenue to refine personalized oncology. Moving forward, efforts should focus on elucidating the mechanisms underlying CD70 regulation and function, and promoting the clinical translation of CD70‐targeted strategies to advance its application as a dual‐purpose target in precision oncology.

## AUTHOR CONTRIBUTIONS

Linhui Wang, Xinxin Gan, and Jiatao Hu were involved in the conception and design of the manuscript. Jiatao Hu, Jinxin Li, and Bo Yang contributed to writing the manuscript. Siyi Wang and Kun Qiao participated in the design of the tables and figures. Yi Bao, Yiren Yang, and Fei Guo provided substantial revisions to the manuscript. All authors have read and approved the final manuscript.

## CONFLICT OF INTEREST STATEMENT

The authors declare no conflicts of interest.

## ETHICS APPROVAL AND CONSENT TO PARTICIPATE

Not applicable.

## CONSENT FOR PUBLICATION

Not applicable.

## Data Availability

All data generated or analyzed during this study are included in this published article.
